# New Triterpenes from *Maytenus robusta*: Structural Elucidation Based on NMR Experimental Data and Theoretical Calculations

**DOI:** 10.3390/molecules171113439

**Published:** 2012-11-12

**Authors:** Grasiely F. Sousa, Lucienir P. Duarte, Antônio F. C. Alcântara, Grácia D. F. Silva, Sidney A. Vieira-Filho, Roqueline R. Silva, Djalma M. Oliveira, Jacqueline A. Takahashi

**Affiliations:** 1 Departamento de Química, Instituto de Ciências Exatas, Universidade Federal de Minas Gerais, 31270-901, Belo Horizonte, MG, Brazil; 2 Escola de Farmácia, Universidade Federal de Ouro Preto, 35400-000, Ouro Preto, MG, Brazil; 3 Departamento de Química e Exatas, Universidade Estadual do Sudoeste da Bahia, 45206-191, Jequié, BA, Brazil

**Keywords:** *Maytenus robusta*, pentacyclic triterpenes, NMR, DFT calculations, acetylcholinesterase inhibitory activity

## Abstract

Leaves of *Maytenus robusta* (Celastraceae) were subjected to phytochemical investigation mainly directed at the isolation of pentacyclic triterpenes. The compounds friedelin (**1**), *β*-friedelinol (**2**), 3-oxo-21*β*-*H*-hop-22(29)-ene (**7**), 3,4-*seco*-friedelan-3,11*β*-olide (**8**), 3*β*-hydroxy-21*β*-*H*-hop-22(29)-ene (**9**), 3,4-*seco*-21*β*-*H*-hop-22(29)-en-3-oic acid (**10**), 3,4-*seco*-friedelan-3-oic acid (**11**), and sitosterol were identified in the hexane extract of *M. robusta* leaves. Compounds **8** and **9** are described herein for the first time. The structure and stereochemistry of both compounds were experimentally established by IR, HRLC-MS, and 1D (^1^H, ^13^C, and DEPT 135) and 2D (HSQC, HMBC and COSY) NMR data and supported by correlations with carbon chemical shifts calculated using the DFT method (BLYP/6-31G* level). Compounds **7** and **10** are also described for the first time, and their chemical structures were established by comparison with NMR data of similar structures described in the literature and correlations with BLYP/6-31G* calculated carbon chemical shifts. Compound **9**, a mixture of **11** and sitosterol, and 3*β*,11*β*-dihydroxyfriedelane (**4**) were evaluated by the Ellman’s method and all these compounds showed acethylcholinesterase inhibitory properties.

## 1. Introduction

Secondary metabolites are isolated from plants and animals, and many of them have been used as sources of derivatives with a large spectrum of biological activities [1], including effects in the treatment of Alzheimer’s disease (AD). AD is a progressive neurodegenerative disorder characterized by a decline in memory and cognitive abilities. About 34 million people around the World have AD, being the major cause of dementia in elderly people [2]. Acetylcholinesterase (AChE) inhibitors are a group of drugs frequently investigated for the symptomatic treatment of AD [3]. Alternatively, the literature also describes some relationships between pentacyclic triterpenes and treatments for AD [4,5,6,7]. 

**Figure 1:**
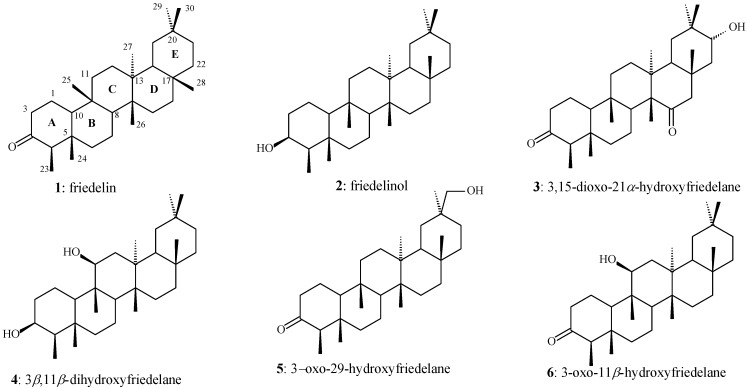
Chemical structure of the triterpenes **1** to **6** previously isolated from the leaves of *M. robusta*.

Some biologically active alkaloids, phenolic compounds, and terpenes have been isolated from some species of *Maytenus* (Celastraceae) [8,9,10,11,12,13,14]. The triterpenes friedelin (3-oxofriedelane; **1**), *β*-friedelinol (3*β*-hydroxyfriedelane; **2**), and 3,15-dioxo-21*α*-hydroxyfriedelane (**3**) were isolated from the leaves of *Maytenus robusta* [15,16,17] (Figure 1). Moreover, we recently studied a white precipitate obtained from the hexane extract of the leaves of *M. robusta*, resulting in isolation and identification of a new triterpene 3*β*,11*β*-dihydroxyfriedelane (**4**) and the known triterpenes **1**, **2**, 3-oxo-29-hydroxyfriedelane (**5**), and 3-oxo-11*β*-hydroxyfriedelane (**6**) [18] (Figure 1). 

Species of the genus *Maytenus* are used in the traditional Brazilian medicine for the treatment of gastric ulcers [19], inflammations, and diarrhea [20], as antimicrobial [21,22], antitumor [23,24], insecticidal agents [25], and for other purposes [14,20]. The antiulcerogenic and antinociceptive activities of *M. robusta* were previously investigated [16,17], but the AChE inhibitory activity of the triterpenes isolated from the species of *Maytenus* has not been tested to date.

**Figure 2:**
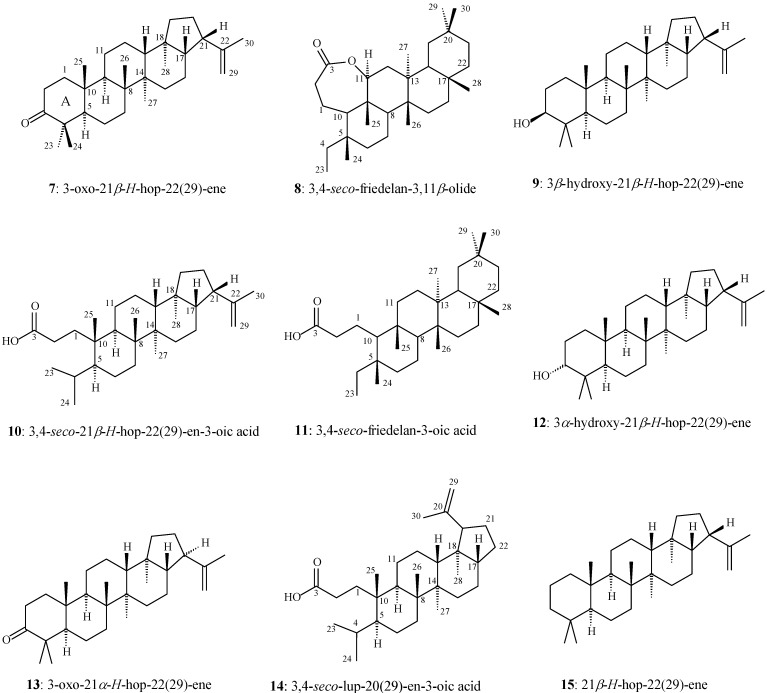
Chemical structure of triterpenes isolated from the leaves of *M. robusta* (compounds **7** to **11**), including similar chemical structures of triterpenes (compounds **12** to **15**) with NMR data described in the literature.

Therefore, the present work describes a phytochemical study of the leaves of *M. robusta* that was directed to the isolation of triterpenes and analysis of their AChE inhibitory activity. The leaf hexane extract of *M. robusta* provided the triterpenes **1**, **2**, 3-oxo-21*β*-*H*-hop-22(29)-ene (**7**), 3,4-*seco*-friedelan-3,11*β*-olide (**8**), 3*β*-hydroxy-21*β*-*H*-hop-22(29)-ene (**9**), 3,4-*seco*-21*β*-*H*-hop-22(29)-en-3-oic acid (**10**), and 3,4-*seco*-friedelan-3-oic acid (**11**) and the steroid sitosterol (Figure 2). 

Triterpenes **8** and **9** are described for the first time in the literature. Their structure and stereochemistry were deduced from experimental IR, HRLC-MS, and 1D (^1^H, ^13^C, and DEPT 135) and 2D (HSQC, HMBC, and COSY) NMR analyses and theoretical methodology based on carbon chemical shifts calculated using the BLYP/6-31G* level of theory.

Compounds **7** and **10** are also new in the literature and were isolated as binary mixtures with **1** and **11**, respectively (Figure 2). The chemical structure of **7** and **10** were established based on 1D NMR analyses, comparison with the NMR spectral data of the terpenes 3*α*-hydroxy-21*β*-*H*-hop-22(29)-ene (**12**) [26], 3-oxo-21*α*-*H*-hop-22(29)-ene (**13**) [27], 3,4-*seco*-lup-20(29)-en-3-oic acid (**14**) [28], and 21*β*-*H*-hop-22(29)-ene (**15**) [26] (Figure 2), and correlations with carbon chemical shifts calculated using BLYP/6-31G*. The chemical structure of compounds **1**, **2**, **11** and sitosterol were based on comparisons with the NMR data available in the literature. The triterpenes **4**, **9**, and **11** were submitted to the Ellman’s bioassay [29,30] and exhibited AChE inhibitory properties.

## 2. Results and Discussion

### 2.1. Structural Analysis

#### 2.1.1. Compounds **1** and **7**

The hexane extract (HE) fractions eluted with 9:1 hexane-chloroform provided a white solid. The IR spectrum of this solid shows two intense absorptions at 1,714 and 1,701 cm^−1^ which are attributed to carbonyl groups. The ^13^C-NMR spectrum shows two groups of 30 signals, each with significant differences in intensity. The ^13^C-NMR data of the group of high-intensity signals (named triterpene **1**) present a signal at *δ*_C_ 213.2 which is characteristic of a carbonyl carbon. The NMR data are similar to the corresponding ones described in the literature for the triterpene friedelin [31]. In turn, the ^13^C-NMR data of the group of low-intensity signals (named triterpene **7**) present a signal at *δ*_C_ 218.2 which is also characteristic of a carbonyl carbon. Two other signals at *δ*_C_ 148.6 and 110.1 (non-hydrogenated and methylene carbon atoms, respectively) are characteristic of alkenyl carbon atoms. The ^1^H-NMR data show a signal at *δ*_H_ 4.78 (integrated for two hydrogen atoms) which is also characteristic of an alkenyl group. These signals are in agreement with a hopane-type skeleton containing a carbonyl carbon. The ^13^C-NMR data of **7** were compared with the hopane-type skeleton data compiled in the literature for **12** and **13** (see Figure 2 and Table 1). Triterpene **7** only differs in relation to the substituent at C-3 of the ring A of **12** and the stereochemistry of the C-21 in the ring E of **13**. In fact, the NMR data of C-1 to C-15 of **13** are very similar to the corresponding data of **7**. On the other hand, the NMR data of C-14 to C-30 (except for C-23 and C-24) of **12** are very similar to the corresponding data of **7**. As result, it can be proposed that the chemical structure of **7** is a combination of the rings A–C of **13** and rings C–E of **12**. The chemical structure of **7** is thus in agreement with that of the compound 3-oxo-21*β*-*H*-hop-22(29)-ene, a triterpene which was not yet described in the literature. Moreover, the intensity and integration of the carbonyl carbon atom signals based on quantitative ^13^C-NMR analysis indicates a mixture 2:1 of compounds **1** and **7**, respectively. BLYP/6-31G* geometry optimization calculations were carried out for **7** with a starting geometry based on the stereochemistry proposed to 3-oxo-21*β*-*H*-hop-22(29)-ene (see Figure 2). The most stable optimized geometry of **7** (*E* = −1246.52767272 a.u.) presents rings A–D in the chair conformation and ring E in the envelope one. Moreover, the C-29 of the allyl group is positioned close to the methyl group at C-28. Carbon chemical shift calculations were carried out on the optimized geometry of **7** at the same level of theory. Correlations between calculated and experimental ^13^C-NMR carbon chemical shift values of the data of **7** (Table 1) provided high correlation coefficient (*R*^2^ = 0.99330) and slope of the *R*^2^ curve (*α* = 0.91728). These theoretical results are also in agreement with the stereochemistry of 3-oxo-21*β*-*H*-hop-22(29)-ene proposed for **7**. 

**Table 1:** ^13^C-NMR data of triterpene **7**, compared with the corresponding data described in the literature for **12** [26] and **13** [27], and ^13^C-NMR data of triterpene **10**, compared with the corresponding data described in the literature for **14** [28] and **15** [26].

Carbon	Compound/ *δ*_C_					
	**7**	**12**	**13**	**10**	**14**	**15**
1	39.6	33.2	39.6	33.8	33.8	40.4
2	34.2	25.4	34.2	28.4	28.3	18.7
3	218.2	76.3	217.9	180.1	180.6	42.1
4	47.4	37.2	47.4	25.4	25.9	33.3
5	54.9	50.1	54.9	40.7	40.7	56.1
6	19.7	18.3	19.8	18.3	25.0	18.7
7	33.7	33.2	32.7	32.0	29.6	33.3
8	41.6	41.9	41.6	40.0	40.5	42.1
9	49.6	49.5	49.7	47.2	47.1	50.4
10	36.8	37.5	36.8	41.5	39.1	37.5
11	21.6	20.9	21.6	21.7	21.5	20.9
12	23.9	23.9	23.9	24.0	24.9	24.0
13	49.6	48.9	48.8	49.6	38.0	49.5
14	42.1	42.1	42.3	42.5	42.9	41.9
15	32.6	33.6	32.7	32.7	27.3	33.6
16	21.6	21.6	20.8	21.8	35.4	21.6
17	54.9	54.9	53.9	54.9	43.1	54.9
18	44.7	44.7	44.2	44.8	48.1	44.8
19	41.9	41.9	40.2	42.0	47.8	41.9
20	27.3	27.4	27.8	27.4	150.6	27.4
21	46.4	46.5	47.9	46.5	29.7	46.5
22	148.6	148.7	148.0	148.7	39.8	148.7
23	26.6	28.6	26.6	19.4	19.5	33.4
24	21.1	22.5	21.1	18.8	18.7	21.6
25	15.7	15.7	15.7	16.5	15.8	15.9
26	16.4	16.6	16.5	24.8	20.0	16.7
27	16.6	16.8	16.5	16.5	14.3	16.7
28	16.1	16.1	15.2	16.2	17.9	16.1
29	110.1	110.1	109.5	110.1	109.3	110.1
30	25.0	25.3	19.7	25.0	19.2	25.0

#### 2.1.2. Compound **2**

The HE fractions eluted with 7:3 hexane-chloroform provided a white solid (named triterpene **2**). The IR spectrum shows an absorption at 3471 cm^−1^, which is attributed to a hydroxyl group. The absorptions at 1384 and 1172 cm^−1^ can be attributed to the asymmetric and symmetric C–O stretches, respectively. The ^1^H-NMR and ^13^C-NMR spectra shows a large signal at *δ*_H_ 3.74 and a signal at *δ*_C_ 72.8 which are characteristic of a carbinolic carbon. The NMR data are similar to the corresponding ones described in the literature for the triterpene *β*-friedelinol [32].

#### 2.1.3. Compound **8**

The HE fractions eluted with 3:2 hexane-chloroform provided a white solid with molecular formula C_30_H_50_O_2_ as deduced from HR-APCIMS (*m/z* 443.3936 [M+H]^+^, calc. 443.3922), named triterpene **8**. The IR spectrum of **8** shows absorptions at 1726, 1288, and 1024 cm^−1^ (attributed to the C=O and asymmetric and symmetric C–O stretches, respectively) which are characteristic of a lactone group. The ^1^H-NMR spectrum of **8** shows a double-doublet signal at *δ*_H_ 4.25 (*J* = 11.2 and 5.2 Hz, integrating for one hydrogen atom) which is characteristic of a hydrogen bonded to an oxygenated sp^3^ carbon and neighboring a methylene carbon in a cyclic system. The multiplet signal at *δ*_H_ 2.65–2.49 (integrating for two hydrogen atoms) can be attributed to the diastereotopic hydrogen atoms of a methylene group that is bonded to a carbonyl group and methylene group in a cyclic system. The seven singlet signals at *δ*_H_ 1.18, 1.09, 1.04, 0.99, 0.97, 0.95, and 0.79 can be attributed to methyl groups bonded to non-hydrogenated carbon atoms. The triplet signal at *δ*_H_ 0.78 (*J* = 7.4 Hz) can be attributed to a methyl group bonded to a methylene carbon. The ^13^C-NMR spectrum of **8** shows a non-hydrogenated carbon signal at *δ*_C_ 175.6 which is attributed to the carbonyl carbon of a lactone group. The signal at *δ*_C_ 84.1 can be attributed to a methynic sp^3^ carbon bonded to the oxygen of the lactone group. The ^13^C-NMR spectrum also shows six non-hydrogenated (*δ*_C_ 42.9, 40.7, 37.9, 36.8, 30.0, and 28.1), three methynic (*δ*_C_ 58.2, 52.6, and 42.6), 11 methylenic (*δ*_C_ 39.2, 38.8, 37.6, 36.1, 35.9, 35.3, 34.5, 32.7, 32.1, 19.1, and 18.0), and eight methylic (*δ*_C_ 34.9, 31.7, 32.0, 22.1, 19.9, 19.3, 13.6, and 7.7) carbon atoms. The COSY contour map of **8** shows correlations of the signal at *δ*_H_ 2.62 (H-2*β*) with the signal at *δ*_H_ 1.73 (H-1*α*); the signal at *δ*_H_ 4.25 (H-11) with the signals at *δ*_H_ 1.67 (H-12*β*) and 1.61 (H-12*α*); the signal at *δ*_H_ 1.48 (H-21*α*) with the signal at *δ*_H_ 0.97 (H-22*β*); and the signal at *δ*_H_ 0.78 (H-23) with the signals at *δ*_H_ 1.32 (H-4*β*) and 1.18 (H-4*α*). The HMBC contour map shows correlations of the hydrogen signals at *δ*_H_ 1.73 (H-1*α*) and 1.58 (H-1*β*) with the carbon signals at *δ*_C_ 175.6 (C-3), 58.2 (C-10), and 34.5 (C-2). The hydrogen signals at *δ*_H_ 2.62 (H-2*β*) and 2.52 (H-2*α*) correlate with the carbon signals at *δ*_C_ 175.6 (C-3), 58.2 (C-10), and 19.1 (C-1). The hydrogen signal at *δ*_H_ 4.25 (H-11) correlates with the carbon signals at *δ*_C_ 58.2 (C-10), 37.6 (C-12), and 13.6 (C-25). The hydrogen signals at *δ*_H_ 1.67 (H-12*β*) and 1.61 (H-12*α*) correlate with the carbon signal at *δ*_C_ 84.1 (C-11). The hydrogen signal at *δ*_H_ 0.78 (H-23) correlates with the carbon signals at *δ*_C_ 36.8 (C-5) and 36.1 (C-4). The hydrogen signal at *δ*_H_ 0.79 (H-24) correlates with the carbon signals at *δ*_C_ 58.2 (C-10), 38.8 (C-6), 36.8 (C-5), and 36.1 (C-4). The hydrogen signal at *δ*_H_ 0.97 (H-25) correlates with the carbon signals at *δ*_C_ 84.1 (C-11), 58.2 (C-10), 52.6 (C-8), and 42.9 (C-9). The NMR data of **8** are in agreement with the data of the triterpene 3,4-*seco*-friedelan-3,11*β*-olide. In fact, the ^13^C-NMR data of **8** were compared with the *seco*-friedelane-type skeleton data compiled in the literature for **11** [33], which only presented significant differences in the functionalities at C-3 and C-11. The NMR data of C-14 to C-23 and C-26 to C-30 of **8** are very similar to the corresponding data of **11** (Table 2). BLYP/6-31G* geometry optimization calculations were carried out for **8** with a starting geometry based on the stereochemistry proposed for 3,4-*seco*-friedelan-3,11-olide (see Figure 2). Two stereochemistry possibilities were considered for carbon C-11: H-11*α* or H-11*β*. 

**Table 2:** NMR data of **8** and corresponding data described in the literature for **11** [33].

Triterpene 8						Compound 11
Atom	Type	δ_C_	δ_H_	HMBC	COSY	δ_C_
1	CH_2_	19.1	1.73 (H *α*); 1.58 (H*β*)	H-2 *α*; H-2*β*		33.2
2	CH_2_	34.5	2.52 (H *α*); *J* = 13.2; t 2.62 (H *β*); *J* = 13.8 and 6.6 Hz; dd	H-1 *α*; H-1*β*	H-1 *α*	25.4
3	C	175.6		H-1α; H-1 *β*; H-2*β*		76.3
4	CH_2_	36.1	1.18 (Ha); 1.32 (Hb)	H-23; H-24		37.2
5	C	36.8		H-23; H-24		50.1
6	CH_2_	38.8	1.38 (H *α* ); 1.59 (H *β*)	H-24		18.3
7	CH_2_	18.0	1.51 (H *α *and H*β*)			33.2
8	CH	52.6	1.34 (H *α*)	H-25; H-26		41.9
9	C	42.9		H-25		49.5
10	CH	58.2	1.25 (H *α*)	H-1 *α*; H-1*β*; H-2*α*; H-2*β*; H-11; H-24; H-25		37.5
11	CH	84.1	4.25 (H *α*); *J* = 5.2 and 11.2 Hz; dd	H-12 *α*; H-12*β*; H-25	H-12	20.9
12	CH_2_	37.6	1.61 (H *α* ); 1.67 (H *β*)	H-11; H-27		23.9
13	C	40.7		H-26; H-27		48.9
14	C	37.9		H-26		42.1
15	CH_2_	32.1	1.54 (H *α* and H*β*)	H-26		33.6
16	CH_2_	35.9	1.39 (H *β* ); 1.56 (H *α*)	H-28		21.6
17	C	30.0				54.9
18	CH	42.6	1.61 (H *β*)	H-27; H-28		44.7
19	CH_2_	35.3	1.39 (H *α* ); 1.24 (H *β*)	H-29; H-30		41.9
20	C	28.1		H-29; H-30		27.4
21	CH_2_	32.7	1.48 (H *α*);	H-29; H-30	H-22 *β*	46.5
22	CH_2_	39.2	1.49 (H *α* ); 0.97 (H *β*)	H-28		148.7
23	CH_3_	7.7	0.78; *J* = 7.4 Hz; t		H-4	28.6
24	CH_3_	22.1	0.79; s			22.5
25	CH_3_	13.6	0.97; s	H-11		15.7
26	CH_3_	19.9	1.04; s			16.6
27	CH_3_	19.3	1.09; s			16.8
28	CH_3_	32.0	1.18; s			16.1
29	CH_3_	34.9	0.95; s	H-30		110.1
30	CH_3_	31.7	0.99; s	H-29		25.3

The optimized geometry of 3,4-*seco*-friedelan-3,11*β*-olide shows lower energy than the optimized geometry of 3,4-*seco*-friedelan-3,11*α*-olide (*E*^electr.−nucl.^ = −1322.96006656 and −1322.94516689 a.u., respectively), corresponding to Δ*E*^electr.−nucl.^ = 9.34 kcal/mol. The geometry of 3,4-*seco*-friedelan-3,11*β*-olide, which does not have the ring A, presents rings B and C in the chair conformation and rings D and E in the boat conformation. Carbon chemical shift calculations were carried out for both the optimized geometries at the same level of theory (BLYP/6-31G*). Correlations between values of calculated carbon chemical shifts and experimental ^13^C-NMR data of **8** (Table 2) provided a higher correlation coefficient and slope (*R*^2^ = 0.98055 and *α* = 0.90931) for 3,4-*seco*-friedelan-3,11*β*-olide than the corresponding values for 3,4-*seco*-friedelan-3,11*α*-olide (*R*^2^ = 0.97441 and *α* = 0.89006). These theoretical results are in agreement with the stereochemistry of 3,4-*seco*-friedelan-3,11*β*-olide proposed for **8**, a triterpene not yet described in the literature.

#### 2.1.4. Compound **9**

The HE fractions eluted with 3:2 hexane-chloroform also provided a white solid with molecular formula C_30_H_50_O as deduced from HR-APCIMS (*m/z* 409.3855 [M+H−18]^+^, calc. 409.3834), named triterpene **9**. The IR spectrum shows an absorption at 3488 cm^−1^ which is characteristic of a hydroxyl group. The weak absorption at 1640 cm^−1^ can be attributed to an alkenyl group. The absorptions at 1372 and 1050 cm^−1^ can be attributed to the asymmetric and symmetric C–O stretches, respectively. The ^1^H-NMR spectrum shows a signal at *δ*_H_ 4.79 (integrating for two hydrogen atoms) which is characteristic of the alkenyl hydrogen atoms of a methylenic carbon. Then, the other alkenyl carbon is non-hydrogenated. The multiplet at *δ*_H_ 3.25–3.21 can be attributed to a carbinolic hydrogen. The multiplet at *δ*_H_ 2.71–2.64 corresponds to a hydrogen neighboring an alkenyl group. The singlets at *δ*_H_ 1.75, 1.02, 0.97, 0.94, 0.83, 0.81, and 0.73 can be attributed to methylic hydrogen atoms. The ^13^C-NMR spectrum shows signals at *δ*_C_ 148.6 (non-hydrogenated carbon) and 110.2 (methylenic carbon) which are characteristic of an alkenyl group. The signal at *δ*_C_ 78.4 is characteristic of a carbinolic carbon. The ^13^C-NMR spectrum also shows other signals which are attributed to five non-hydrogenated (*δ*_C_ 44.8, 42.1, 41.7, 39.0, and 37.2), five methynic (*δ*_C_ 55.3, 54.9, 50.4, 49.5, and 46.5), 10 methylenic (*δ*_C_ 41.9, 38.9, 33.7, 33.4, 27.5, 27.4, 24.0, 21.7, 21.1, and 18.5), and seven methylic (*δ*_C_ 28.2, 25.0, 16.7, 16.6, 16.1, 15.9, and 15.7) carbon atoms (see Table 3). The COSY contour map of **9** shows correlations of the signal at *δ*_H_ 3.23 (H-3) with the signal at *δ*_H_ 1.65 (H-2); the signals at *δ*_H_ 1.53 (H-6*β*) and 1.40 (H-6*α*) with the signal at *δ*_H_ 0.71 (H-5); the signals at *δ*_H_ 1.97 (H-20*β*) and 1.86 (H-20*α*) with the signals at *δ*_H_ 1.61 (H-19*α*) and 1.04 (H-19*β*); the signal at *δ*_H_ 2.67 (H-21) with the signals at *δ*_H_ 1.39 (H-17), 1.97 (H-20*β*), and 1.86 (H-20*α*); the signal at *δ*_H_ 4.79 (H-29) with the signals at *δ*_H_ 2.67 (H-21) and 1.75 (H-30). The HMBC contour map shows correlations of the hydrogen signal at *δ*_H_ 3.23 (H-3) with the carbon signals at *δ*_C_ 39.0 (C-4), 28.2 (C-23), and 15.7 (C-24); the hydrogen signal at *δ*_H_ 2.67 (H-21) with the carbon signals at 148.6 (C-22), 110.2 (C-29), *δ*_C_ 54.9 (C-17), 44.8 (C-18), 27.4 (C-20), and 25.0 (C-30); the hydrogen signal at *δ*_H_ 4.79 (H-29) with the carbon signals at 148.6 (C-22), *δ*_C_ 46.5 (C-21), and 25.0 (C-30); the hydrogen signal at *δ*_H_ 1.75 (H-30) with the carbon signals at 148.6 (C-22), 110.2 (C-29), and *δ*_C_ 46.5 (C-21).

**Table 3:** NMR data of triterpene **9** and corresponding data described in the literature for **12** [26].

Triterpene 9						Compound 12
Atom	Type	δ_C_	δ_H_	HMBC	COSY	δ_C_
1	CH_2_	38.9	0.94 (H *α*);1.70 (H*β*)	H-25		33.2
2	CH_2_	27.5	1.63 (H *α* and H*β*)			25.4
3	CH	78.4	3.23 (H *α*); m	H-23; H-24	H-2	76.3
4	C	39.0		H-3; H-23; H-24		37.2
5	CH	55.3	0.69 (H *α*)	H-23; H-24; H-25		50.1
6	CH_2_	18.5	1.40 (H *α*); 1.53 (H*β*)		H-5	18.3
7	CH_2_	33.4	1.47 (H *α*); 1.62 (H*β*)	H-26		33.2
8	C	41.7		H-26; H-27		41.9
9	CH	50.4	1.24 (H *α*)	H-25; H-26		49.5
10	C	37.2		H-5; H-25		37.5
11	CH_2_	21.1	1.51 (H *α*); 1.32 (H*β*)			20.9
12	CH_2_	24.0	1.43 (H *α*); 1.49 (H*β*)			23.9
13	CH	49.5	1.37 (H *β*)	H-27; H-28		48.9
14	C	42.1		H-26; H-27		42.1
15	CH_2_	33.7	1.42 (H *α*); 1.24 (H*β*)	H-27		33.6
16	CH_2_	21.7	1.74 (H *α*); 1.65 (H*β*)			21.6
17	CH	54.9	1.39 (H *β*)	H-21; H-28		54.9
18	C	44.8		H-21; H-28		44.7
19	CH_2_	41.9	1.60 (H *α*); 1.04 (H*β*)	H-28		41.9
20	CH_2_	27.4	1.84 (H *α*); 1.97 (H*β*)	H-21	H-19	27.4
21	CH	46.5	2.67 (H *β*); *J* = 16.6 and 9.0 Hz; dd	H-29; H-30	H-17; H-20	46.5
22	C	148.6		H-21; H-29; H-30		148.7
23	CH_3_	28.2	1.02; s	H-3; H-24		28.6
24	CH_3_	15.7	0.81; s	H-3; H-23		22.5
25	CH_3_	16.7	0.83; s			15.7
26	CH_3_	15.9	0.94; s			16.6
27	CH_3_	16.7	0.97; s			16.8
28	CH_3_	16.1	0.73; s			16.1
29	CH_2_	110.2	4.79; s	H-21; H-30	H-30; H-21	110.1
30	CH_3_	25.0	1.75; s	H-21; H-29		25.3

The NMR analyses of **9** are in agreement with the data of the triterpene 3*β*-hydroxy-21*β*-*H*-hop-22(29)-ene. In fact, the ^13^C-NMR data of **9** were compared with the hopane-type skeleton data compiled in the literature for **12** [26], and seen to only present a significant difference in the stereochemistry at C-3. The NMR data of C-6 to C-8, C-10 to C12, C-14 to C-23, and C-27 to C-30 of **9** are very similar to the corresponding data of **12** (Table 3). BLYP/6-31G* geometry optimization calculations were carried out for **9** with a starting geometry based on the stereochemistry proposed to 3*β*-hydroxy-21*β*-*H*-hop-22(29)-ene (Figure 2). The most stable optimized geometry (*E* = −1247.70633974 a.u.) presents the rings A, B, C, and D in the chair conformation and the ring E in the envelope one. Moreover, the C-29 of the allyl group is positioned close to the methyl group at C-28. Carbon chemical shift calculations were carried out to the optimized geometry of **9** at the same level of theory (BLYP/6-31G*). Correlations between values of calculated carbon chemical shifts and experimental ^13^C-NMR data of **9** (Table 3) provided a high correlation coefficient (*R*^2^ = 0.98817) and slope of the *R*^2^ curve (*α* = 0.93702). These theoretical results are in agreement with the stereochemistry of 3*β*-hydroxy-21*β*-*H*-hop-22(29)-ene for **9**, a triterpene not yet described in the literature.

#### 2.1.5. Compounds **10** and **11**

The HE fractions eluted with 1:1 hexane-chloroform provided a white solid. The IR spectrum of the solid shows a large absorption at 3250–2700 cm^−1^ and an intense absorption at 1701 cm^−1^ which are characteristic of a carboxylic acid group. Moreover, the absorptions at 1284 and 1049 cm^−1^can be attributed to the asymmetric and symmetric C–O stretches, respectively. The ^1^H-NMR spectrum shows a broad signal at *δ*_H_ 4.78 (integrating for two hydrogen atoms) which is characteristic of an alkenyl group. The ^13^C-NMR spectrum shows two groups of 30 signals, each with significant differences in intensity. The ^13^C-NMR data of the group of low-intensity signals (named triterpene **10**) present a signal at *δ*_C_ 180.1 which is characteristic of a carboxylic carbon. The signals at *δ*_C_ 148.7 and 110.1 (non-hydrogenated and methylenic carbon atoms, respectively) are characteristic of an alkenyl group. The ^13^C-NMR data of **10** were compared with the corresponding data compiled in the literature for **14** and **15** (see Table 1). Triterpene **10** only differs in the position of the allyl group and the opening of the ring A in relation to **14** and **15**, respectively (see Figure 2). The NMR data of C-1 to C-5, C-8, C-9, and C-11 of **14** are very similar to the corresponding data of **10**. On the other hand, the NMR data of the C-12 to C-22 of **10** are very similar to the corresponding data of **15**. As result, it can be proposed that the chemical structure of **10** is in agreement with the structure of the triterpene 3,4-*seco*-21*β*-*H*-hop-22(29)-en-3-oic acid, a triterpene which was not yet described in the literature. BLYP/6-31G* geometry optimization calculations were carried out for **10** with starting geometry based on the stereochemistry proposed for 3,4-*seco*-21*β*-*H*-hop-22(29)-en-3-oic acid (see Figure 2). The most stable optimized geometry (*E* = −1322.93835876 a.u.), which does not have the ring A, presents the rings B, C, and D in the chair conformation and ring E in the envelope one. Carbon chemical shift calculations were carried out to the optimized geometry of **10** at the same level of theory (BLYP/6-31G*). Correlations between values of calculated carbon chemical shifts and experimental ^13^C-NMR data of **10** (Table 1) provided high correlation coefficient (*R*^2^ = 0.97833) and slope of the *R*^2^ curve (*α* = 0.87424). These theoretical results are in agreement with the stereochemistry of 3,4-*seco*-21*β*-*H*-hop-22(29)-en-3-oic acid proposed for **10**, a triterpene not yet described in the literature. In turn, the ^13^C-NMR data of the group of high intensity signals (named triterpene **11**) also presents a signal characteristic of carboxyl carbon (at *δ*_C_ 178.2). The ^13^C-NMR data of **11** are similar to the corresponding data described in the literature for 3,4-*seco*-friedelan-3-oic acid [33]. Moreover, the intensity and integration of the carbonyl carbon atom signals based on quantitative ^13^C-NMR analysis indicates a mixture 2:3 of compounds **10** and **11**, respectively.

### 2.2. *In Vitro* AChE Inhibitory Activity

The AChE activity was measured for the triterpenes **4**, **9**, and mixture of **11** and sitosterol which were previously obtained from the leaves of *M*. *robusta*. The calorimetric method of Ellman was adapted for 96-well microplates in the assays at 25 °C [30]. The triterpenes **4** and **9** showed (64 ± 3)% and (76 ± 1)% of inhibition, respectively. The mixture of triterpene **11** and sitosterol exhibited very significant results, *i.e.*, (94 ± 1)% of inhibition.

## 3. Experimental

### 3.1. General Procedures

Uncorrected melting points were determined using a Microquímica apparatus, model MQAPF-302. Optical rotations were measured on a Perkin-Elmer Model 341 polarimeter using a 100 mm, 1.0 mL capacity cell. The IR spectra were taken on a Perkin Elmer-Spectrum One (ATR) spectrometer. The ^1^H and ^13^C-NMR spectra at 400.129 and 100.613 MHz, respectively, as well as the COSY, HSQC, and HMBC experiments were performed on a Brüker DRX400 AVANCE spectrometer, using CDCl_3_ or a mixture of CDCl_3_/pyridine-d_5_ as solvent, with direct or inverse probes and a field gradient. The chemical shifts were registered in ppm (*δ*) relative to TMS as the internal standard. The coupling constants (*J*) were registered in Hertz. HR-APCIMS spectra were acquired on a Shimadzu LCMS-IT-TOF system. Analyses were carried out using manual injection. The samples were dissolved in CHCl_3_ and then diluted with MeOH. Column chromatography (CC) processes were carried out using silica gel 60 (70–230 Mesh). Thin layer chromatography (TLC) processes were carried out using precoated silica gel plates. 

### 3.2. Phytochemical Methodology

#### 3.2.1. Plant Material

Leaves of *M. robusta* were collected in June 2010 at the Parque Estadual do Itacolomi, in the City of Ouro Preto, State of Minas Gerais, Brazil. After botanical identification, the voucher specimen of *M. robusta* was deposited in the Herbário Professor José Badini, Universidade Federal de Ouro Preto, under the code OUPR: 25,559.

#### 3.2.2. Extraction and Isolation of Constituents

Leaves of *M. robusta* were dried at room temperature until a constant weight was achieved (about one week) and finally powdered. A sample of this material (864.4 g) was submitted to extraction with hexane (3 L, 5 days, room temperature). A solid material (**SM**; 4.51 g) precipitated during solvent evaporation, being separated by filtration under reduced pressure. The **SM** was submitted to column chromatography using silica gel as the stationary phase (CCS) eluted with hexane, chloroform, ethyl acetate, and methanol in increasing polarity order. The triterpenes **1**–**6** (Figure 1) were obtained, as previously reported [18].

The rest of the hexane extract provided a viscous crude oil (**HE**; 32.0 g) after complete solvent evaporation. A part of **HE** (31.43 g) was submitted to CCS eluted with hexane, chloroform, ethyl acetate, and methanol in increasing polarity order. The **HE** fractions eluted with hexane-chloroform (9:1) were again submitted to CCS eluted with hexane and chloroform in increasing polarity order. The fractions eluted with hexane-chloroform (1:1) provided a white solid (13.5 mg) which was identified as a mixture of the triterpenes **1** and **7**. The **HE** fractions eluted with hexane-chloroform (4:1) provided a white solid (624.0 mg) which was identified as triterpene **1**. The **HE** fractions eluted with hexane-chloroform (7:3) provided a solid (566.1 mg) which was identified as triterpene **2**. 

The **HE** fractions eluted with hexane-chloroform (3:2) were again submitted to CCS eluted with hexane, chloroform, ethyl acetate, and methanol in increasing polarity order. The fractions hexane-chloroform (3:7) provided a white solid (14.1 mg) which was identified as triterpene **8**. The fractions eluted with chloroform (289.0 mg) were submitted to CCS eluted with chloroform, providing a white solid (103.0 mg) which was identified as triterpene **9**.

The **HE** fractions eluted with hexane-chloroform (1:1) were again submitted to CCS eluted with hexane, chloroform, ethyl acetate, and methanol in increasing polarity order. The fractions eluted with hexane-chloroform (3:7) provided a white solid (59.5 mg) which was identified as a mixture of the triterpenes **10** and **11**. The fractions eluted with hexane-chloroform (1:9) provided a white solid (83.8 mg) which was identified as a mixture of **11** and the steroid sitosterol.

*Friedelin* (**1**): white solid (624.0 mg); m.p. 251–254 °C; IR (ATR; cm^−1^) *ν* 2972, 2926, 2868, 1711, 1461, 1389, 1299, 1189, 1073, 1002, 982, and 924; ^1^H-NMR (400 MHz; CDCl_3_; ppm) *δ*_H_ 2.42–2.40 (multiplet; 1H), 2.38–2.37 (multiplet; 2H), 1.97–1.94 (multiplet; 1H), 1.77–1.34 (superposed signals; 21H), 1.29 (s; 3H), 1.18 (s; 3H), 1.05 (s; 3H), 1.01 (s; 3H), 0.95 (s; 3H), 0.89 (d, *J* = 6.4 Hz; 3H), 0.87 (s; 3H), and 0.73 (s; 3H); ^13^C-NMR (100 MHz; CDCl_3_; ppm) *δ*_C_ 213.2 (C-3), 59.5 (C-10), 58.2 (C-4), 53.1 (C-8), 42.8 (C-18), 42.2 (C-5), 41.5 (C-2), 41.3 (C-6), 39.7 (C-14), 39.3 (C-22), 38.3 (C-13), 37.5 (C-9), 36.0 (C-16), 35.6 (C-11), 35.4 (C-19), 35.0 (C-29), 32.8 (C-21), 32.4 (C-15), 32.1 (C-28), 31.8 (C-30), 30.5 (C-12), 30.0 (C-17), 28.2 (C-20), 22.3 (C-1), 20.3 (C-26), 18.7 (C-27), 18.3 (C-7), 18.0 (C-25), 14.7 (C-24), and 6.8 (C-23). HR-APCIMS (*m/z* 427.3969 [M+H]^+^, calc. 427.3934).

*β-Friedelinol* (**2**): white solid (566.1 mg); m.p. 271–276 °C; IR (ATR; cm^−1^) *ν* 3619, 3471, 2915, 2869, 1448, 1384, 1360, 1172, 1089, 1020, 1000, 979, and 920; ^1^H-NMR (200 MHz; CDCl_3_; ppm) *δ*_H_3.74 (ls; H-3; 1H) and 2.50–0.80 (superposed signals); ^13^C-NMR (50 MHz; CDCl_3_; ppm) *δ*_C_ 72.8 (C-3), 61.3 (C-10), 53.2 (C-8), 49.1 (C-4), 42.8 (C-18), 41.7 (C-6), 39.7 (C-14), 39.3 (C-22), 38.4 (C-13), 37.8 (C-5), 37.1 (C-9), 36.1 (C-2), 35.5 (C-16), 35.3 (C-11), 35.2 (C-19), 35.0 (C-29), 32.8 (C-21), 32.3 (C-15), 32.1 (C-28), 31.8 (C-30), 30.6 (C-12), 30.0 (C-17), 28.2 (C-20), 20.1 (C-26), 18.6 (C-27), 18.2 (C-25), 17.5 (C-7), 16.4 (C-24), 15.8 (C-1), and 11.6 (C-23); HR-APCIMS (*m/z* 411.3966 [M+H−18]^+^, calc. 411.3985). 

*3-Oxo-21β-H-hop-22(29)-ene* (**7**): white solid (13.5 mg) obtained in mixture with **1**; ^1^H-NMR (200 MHz; CDCl_3_; ppm) *δ*_H_ 4.78 (ls; 2H) and 2.43–0.73 (superposed signals). The ^13^C-NMR data of **7** are shown in Table 1.

*3,4-seco-Friedelan-3,11β-olide* (**8**): white solid (14.1 mg); m.p. 184–187 °C; IR (ATR; cm^−1^) 2962, 2850, 1726, 1458, 1386, 1288, and 1024; ^1^H (400 MHz; CDCl_3_; ppm) *δ*_H_ 4.25 (dd, *J* = 11.2 and 5.2 Hz; H-11*α*), 2.62 (dd, *J* = 13.8 and 6.6 Hz; H-2*β*), 2.52 (t, *J* = 13.2 Hz; H-2*α*), 1.73 (H-1*α*), 1.67 (H-12*β*), 1.61 (H-12*α* and H-18*β*), 1.59 (H-6*β*), 1.58 (H-1*β*), 1.56 (H-16*α*), 1.54 (H-15*α* and H-15*β*), 1.51 (H-7*α* and H-7*β*), 1.49 (H-22*α*), 1.48 (H-21*α*), 1.39 (H-16*β*, H-17*β*, and H-19*α*), 1.38 (H-6*α*), 1.34 (H-8*α*), 1.32 (H-4b), 1.30 (H-21*β*), 1.25 (H-10*α*), 1.24 (H-19*β*), 1.18 (H-4a and H-28), 1.09 (H-27), 1.04 (H-26), 0.99 (H-30), 0.97 (H-22*β* and H-25), 0.95 (H-29), 0.79 (H-24), and 0.78 (t, *J* = 7.4 Hz; H-23); the ^13^C-NMR data of **8** are shown in Table 2; HR-APCIMS (*m/z* 443.3936 [M+H]^+^, calc. 443.3922).

*3β-Hydroxy-21β-H-hop-22(29)-ene* (**9**): white solid (103.0 mg); m.p. 217–221 °C; [*α*]^2^°_D_= +46 (*c* = 2.22 × 10^−3^ M; CHCl_3_); IR (ATR; cm^−1^) *ν* 3488, 2931, 2870, 1640, 1445, 1372, 896, and 886; ^1^H-NMR (400 MHz; CDCl_3_/pyridine-d_5_; ppm) *δ*_H_ 4.79 (s; H-29), 3.23 (m; H-3*α*), 2.67 (dd, *J* = 16.6 and 9.0 Hz; H-21), 1.97 (H-20*β*), 1.84 (H-20*α*), 1.75 (H-30), 1.74 (H-16*α*), 1.70 (H-1*β*), 1.65 (H-16*β*), 1.63 (H-2*α* and H-2*β*), 1.62 (H-7*β*), 1.60 (H-19*α*), 1.53 (H-6*β*), 1.51 (H-11*α*), 1.49 (H-12*β*), 1.47 (H-7*α*), 1.43 (H-12*α*), 1.42 (H-15*α*), 1.40 (H-6*α*), 1.39 (H-17*β*), 1.37 (H-13*β*), 1.32 (H-11*β*), 1.24 (H-9*α* and H-15*β*), 1.04 (H-19*β*), 1.02 (H-23), 0.97 (H-27), 0.94 (H-1*α* and H-26), 0.83 (H-25), 0.81 (H-24), 0.73 (H-28), and 0.69 (H-5*α*); the ^13^C-NMR data of **9** are shown in Table 3; HR-APCIMS (*m/z* 409.3855 [M+H−18]^+^, calc. 409.3834). 

*3,4-seco-21β-H-Hop-22(29)-en-3-oic acid* (**10**): white solid obtained as a mixture with **11** (59.5 mg); ^1^H-NMR (400 MHz; CDCl_3_; ppm) *δ*_H_ 4.78 (ls; 2H), 2.67 (dd, *J* = 16.4 and 9.6 Hz), 2.38 (t, *J* = 8.7 Hz), 1.75–0.73 (superposed signals). The ^13^C-NMR data of **10** are shown in Table 1.

*3,4-seco-Friedelan-3-oic acid* (**11**) [33]: white solid obtained in mixture with **10** (59.5 mg); ^1^H-NMR (400 MHz; CDCl_3_; ppm) *δ*_H_ 2.38 (t, *J* = 8.7 Hz) and 1.75–0.73 (superposed signals). The ^13^C-NMR data of **11** are shown in Table 2.

### 3.3. Theoretical Methodology

Theoretical studies were carried out using the Gaussian 03 software package [34]. The geometries obtained from PM3 semi-empirical calculations were used as initial models in geometry optimizations employing DFT calculations with the Pople’s split valence basis set 6-31G*. BLYP exchange-correlation functional was used in DFT calculations. The optimized geometries were characterized as true minima on the potential energy surface (PES) when all harmonic frequencies were real. The electronic-nuclear energy (*E*) of the optimized geometries was given in atomic unit (Hartree). This theoretical methodology has been efficiently employed in the study of different organic compounds, including terpenes [35,36,37,38].

The optimized geometries were used to calculate carbon chemical shifts at the same levels of theory. Values of calculated carbon chemical shift (*σ*_C_) were determined in relation to the corresponding calculated value for tetramethylsilane (*σ*_C_ 187.97). Correlations between *σ*_C_ values and experimental carbon chemical shifts (*δ*_C_) were obtained using software package Origin™ Standard 7.5. The *σ*_C_ and *δ*_C_ values were plotted on the *x* and *y* axes, respectively. The *σ*_C_/*δ*_C_ correlation curves were given as linear fits with correlation coefficients (*R*^2^) and slope of the *R*^2^ curve (*α*) furnished by the program. The BLYP/6-31G* calculations usually give satisfactory results of carbon chemical shifts, as have been obtained in previous works [39,40,41].

### 3.4. *In Vitro* AChE Inhibitory Activity

The buffers A (50 mM Tris–HCl, pH 8, containing 0.1 M NaCl and 0.02 M MgCl_2_.6H_2_O), B (50 mM Tris–HCl, pH 8, containing 0.1% bovine serum albumin), and C (50 mM Tris–HCl, pH 8) were prepared to study the *in vitro* AChE inhibitory activity. This activity was measured using a 96-well microplate reader based on an adapted Ellman’s method [29,30]. The enzyme hydrolyzes the substrate acethylthiocholine. The obtained product, thiocholine, decomposes the Ellman’s reagent, 5,5-dithiobis-(2-nitrobenzoic acid) (DTNB), providing 2-nitrobenzoate-5-mercaptothiocholine and 5-thio-2-nitrobenzoate, which can be detected at 405 nm.

Volumes of acetylthiocholine iodide (25 μL, 15 mM in water), DTNB (125 μL, 3 mM in buffer A), buffer B (50 μL), and sample (25 μL, 10 mg/mL in MeOH diluted 10-fold with buffer C, resulting in a concentration of 1 mg/mL) were added into each well of a 96-well microplate. Instead of adding the sample solution, a volume of 25 μL of buffer C was employed to prepare the blank sample. The positive control was prepared under the same conditions, using physostigmine (eserine) as standard. Tests were carried out in quintuplicate. The absorbance was measured at 405 nm every 60 s by eight times using a Elisa Thermoplate microplate reader. After addition of 25 μL of acetylcholinesterase solution (0.226 U/mL in buffer B), the absorbance was again read every 60 s for ten times. The increase in absorbance relative to substrate spontaneous hydrolysis was corrected by reaction rate variation before and after addition of the enzyme. The inhibition percentage was calculated by comparing the rates of the sample with the blank.

## 4. Conclusions

The hexane extract of the leaves of *M. robusta* provided seven triterpenes. The triterpenes **1**, **2**, and **11** were also isolated in a previous phytochemical investigation. The triterpenes **8** and **9** are described for the first time in the literature. The triterpenes **7** and **10** are also new compounds, but both compounds were obtained as a mixture. Hopane and *seco*-hopane triterpenoids are not usual in species of the family Celastraceae. The combination of experimental NMR analyses with carbon chemical shift calculations was a useful procedure for the structural determination of these hopane and friedelane triterpenes. Compounds **4**, **9**, and the mixture of **11** and sitosterol showed acetylcholinesterase inhibitory properties. These compounds present hopane- and friedelane-type skeletons, suggesting biological potential of their derivatives for Alzheimer’s desease.

## Supplementary Material

Figures with liner fit curves obtained from correlations between experimental and calculated carbon chemical shifts are shown as Supplementary Material, which can be accessed at: http://www.mdpi.com/1420-3049/17/11/13439/s1. Tables with the geometric parameters and other results of all the optimized structures considered in this work are available from the authors upon request.
