# Supplementary material for: New Triterpenes from Maytenus robusta: Structural Elucidation Based on NMR Experimental Data and Theoretical Calculations
Source: Molecules. 2012 Nov 12;17(11):13439–56. doi: 10.3390/molecules171113439 (PMC6268669; doi:10.3390/molecules171113439)

Supplementary Information

Figure S1. Correlation between the BLYP/6-31G* calculated carbon chemical shifts and the corresponding experimental ^{13}C-NMR data of **7**.

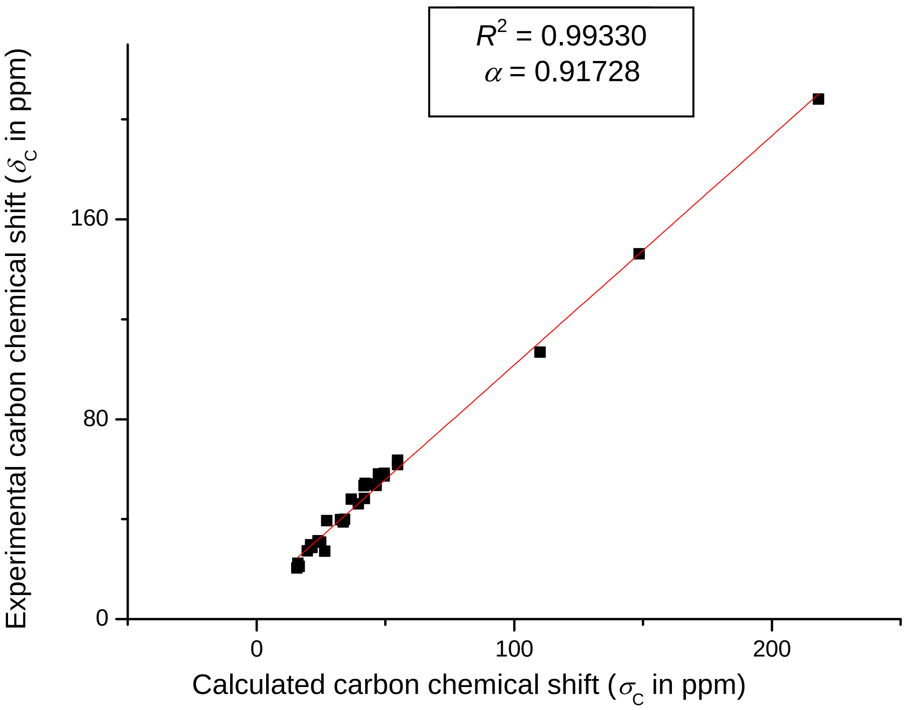

Figure S2. Correlation between the BLYP/6-31G* calculated carbon chemical shifts and the corresponding experimental ^{13}C-NMR data of **8**.

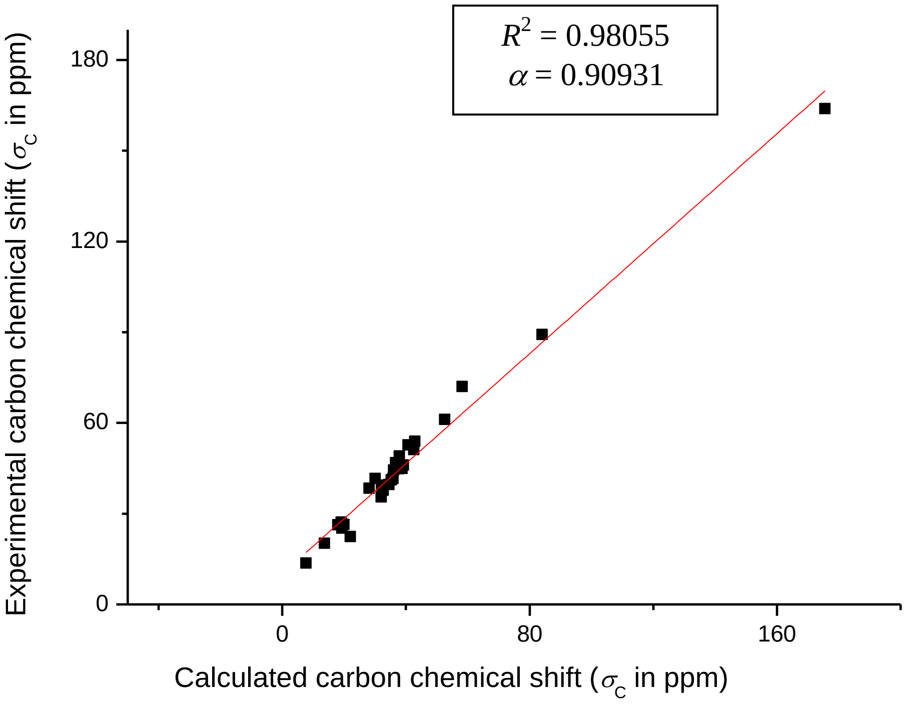

Figure S3. Correlation between the BLYP/6-31G * calculated carbon chemical shifts and the corresponding experimental ^{13}C-NMR data of **9**.

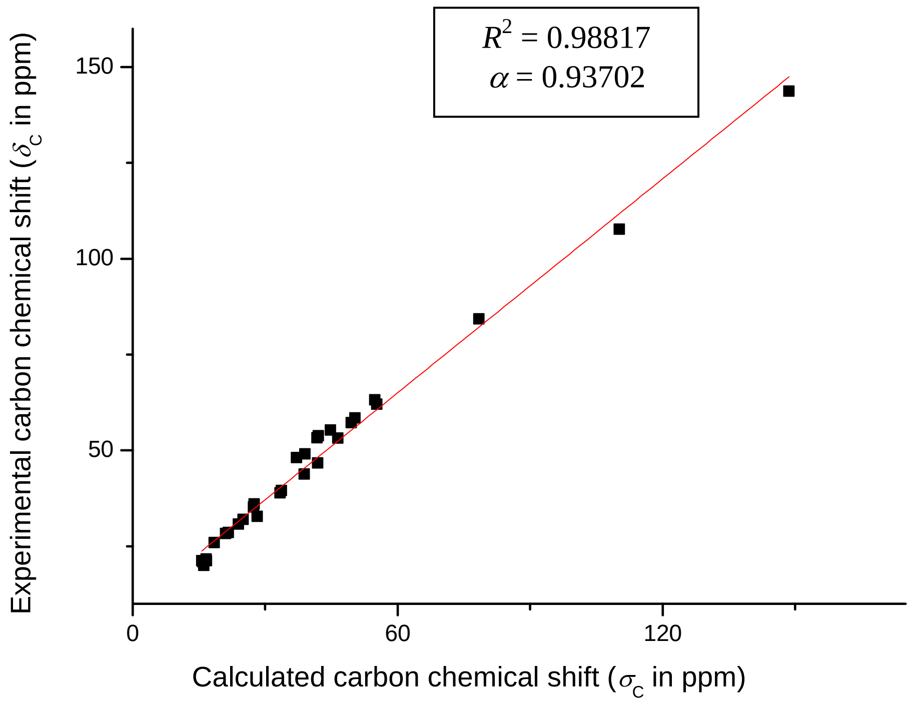

Figure S4. Correlation between the BLYP/6-31G* calculated carbon chemical shifts and the corresponding experimental ^{13}C-NMR data of **10**.

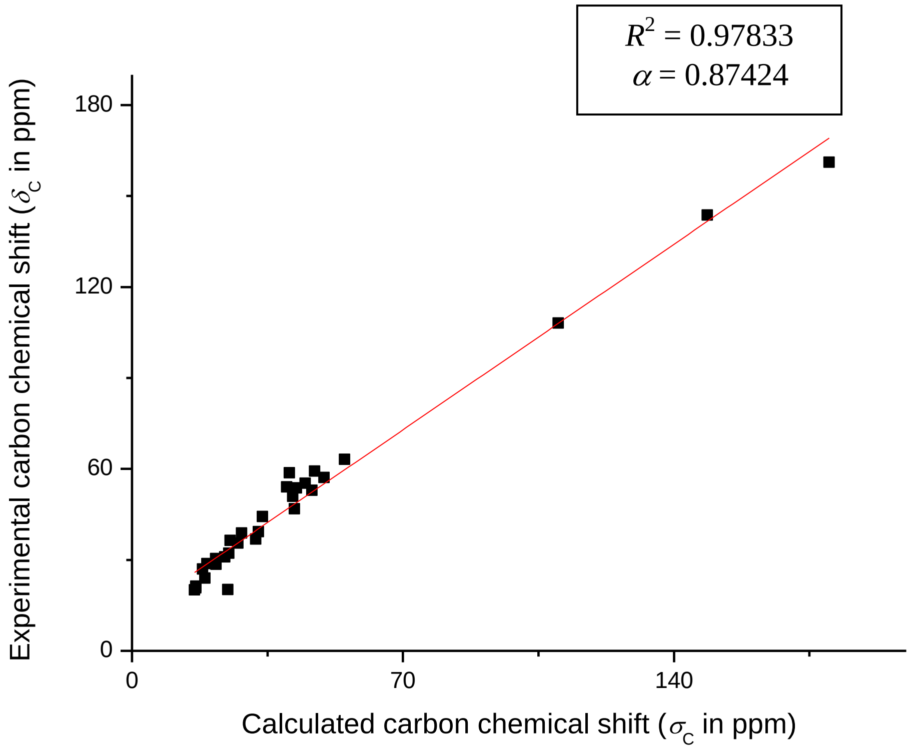

Supplement: Supplementary file 1 [file molecules-17-13439-s001.pdf]
